# Endoscopic Treatment of Complex Walled-Off Necrosis in Necrotizing Pancreatitis With Two Simultaneous Lumen-Apposing Metal Stents: A Case Report

**DOI:** 10.7759/cureus.30930

**Published:** 2022-10-31

**Authors:** Marcos Eduardo Lera dos Santos, Igor Mendonça Proença, João Guilherme Ribeiro Jordão Sasso, Victor Lira de Oliveira, Pedro Henrique Boraschi Vieira Ribas, Alexandre Moraes Bestetti, Igor Braga Ribeiro, Raoni Salomão Sant Anna, Guilherme Henrique Peixoto de Oliveira, Eduardo Guimarães Hourneaux de Moura

**Affiliations:** 1 Serviço de Endoscopia Gastrointestinal do Departamento de Gastroenterologia, Hospital das Clínicas da Faculdade de Medicina da Universidade de São Paulo, São Paulo, BRA; 2 Departamento de Cirurgia do Aparelho Digestivo, Serviço de Cirurgia do Hospital Santa Marcelina, São Paulo, BRA

**Keywords:** pancreatic necrosis, endoscopic ultrasound, endoscopic necrosectomy, lumen‐apposing metal stent, walled-off necroses

## Abstract

Organized pancreatic and peripancreatic collections are complications of pancreatitis and should be treated when symptomatic or complicated. When feasible, the endoscopic ultrasound approach presents high efficacy and low morbidity and mortality, making it the first likely option. Among the available accessories for endoscopic drainage, the lumen-apposing metal stent can be a better option, with a low migration rate; furthermore, it allows endoscopic necrosectomy. Here, we present the case of complex walled-off necrosis treated with two lumen-apposing metal stents in the same procedure.

A 41-year-old male patient with walled-off necrosis presented with delayed gastric emptying and obstruction of the main biliary duct. Magnetic resonance imaging and endoscopic ultrasound revealed two non-communicating collections. We opted for endoscopic ultrasound-guided drainage with the deployment of two simultaneous lumen-apposing metal stents: one transduodenal and the other transgastric, with clinical improvement. After three weeks, endoscopic retrograde cholangiopancreatography showed a biliary fistula communicating with the periduodenal collection, which was treated with a biliary plastic stent. An endoscopic necrosectomy was performed, and the metal stents were removed. Control magnetic resonance imaging demonstrated improvement. The patient was asymptomatic at the six-month follow-up.

The treatment of symptomatic complex walled-off necrosis remains a challenge and may require multiple endoscopic approaches; moreover, surgical treatment may be necessary in case of failure. In the present report, we demonstrate that the deployment of two lumen-apposing metal stents in the same procedure is feasible when necessary as it was associated with technical success and short-term clinical success.

## Introduction

Pancreatic and peripancreatic collections are complications of pancreatitis and should be classified according to the revised Atlanta criteria, considering the evolution time and the characteristics of their content, as it can be fluid or thick/solid [1]. Spontaneous resolution is not uncommon; however, collections that are symptomatic or complicated should be treated [2]. The main symptoms and complications include abdominal pain, delayed gastric emptying, and infection [3]. When technically feasible, endoscopic ultrasound (EUS)-guided drainage is considered the gold standard treatment for chronic pancreatic and peripancreatic collections, for both pseudocyst and walled-off necrosis (WON), due to its high efficiency and low morbidity and mortality associated. In addition, EUS drainage presents certain advantages when compared to surgical treatment [1,3,4].

EUS drainage of pancreatic collections should always be performed under general anesthesia due to the risk of bronchoaspiration. Several stents of different materials, sizes, and shapes are available to carry out EUS drainage. Among the options, we highlight the lumen-apposing metallic stent (LAMS). The LAMS provides better anchorage, possibly reducing the migration rate, and allows access to the collections with the gastroscope through the stent to perform direct endoscopic necrosectomy. Furthermore, the use of the LAMS is safe with high clinical and technical success [5,6]. Among the various options of LAMS, the Hot-AxiosT system [7] is a practical and quick option for EUS drainage with a delivery system that allows the implantation of the stent in a short procedure time. Thus, it appears to be a reasonable option when drainage is indicated with more than one stent.

## Case presentation

A 41-year-old male patient, with a medical history of severe acute pancreatitis secondary to hypertriglyceridemia two months before, presented with symptoms of delayed gastric emptying, a palpable abdominal mass, fever, and cholestatic syndrome. The initial blood parameters are presented in Table 1.

**Table 1:** Initial blood parameters. CRP: C-reactive protein; ALT: alanine aminotransferase; AST: aspartate aminotransferase

Parameter	Value
Hemoglobin	8.3 g/dL
Leukocytes	16,400 cells/mm^3^
Platelets	215,000 cells/mm^3^
Total bilirubin	12.9 mg/dL
Direct bilirubin	9.6 mg/dL
CRP	40.7 mg/dL
Amylase	546 U/L
ALT	382 U/L
AST	277 U/L

**Figure 1:**
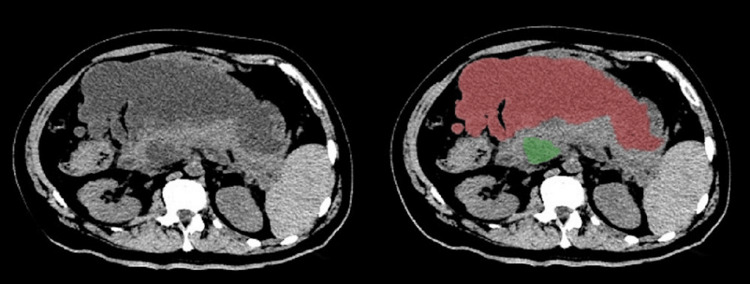
Peripancreatic collections. Abdominal computed tomography showing two peripancreatic collections: anterosuperior (red) and posteroinferior (green).

An abdominal computed tomography (CT) and magnetic resonance imaging (MRI) demonstrated two septate peripancreatic collections, that is, an anterosuperior and a posteroinferior, apparently non-communicating, with heterogeneous content, measuring 26 × 14 × 12 cm. In addition, there was a 15 mm common bile duct (CBD) probably due to extrinsic compression (Figure 1).

The esophagogastroduodenoscopy (EGD) identified a bulge in the posterior gastric wall from the gastric body to the distal antrum, and a second bulge in the duodenum, which included the bulb and the second duodenal portion. EUS was performed that showed two non-communicating collections with heterogeneous content, compatible with WON. Furthermore, extrinsic compression of the CBD by the smaller collection, periduodenal posteroinferior, was revealed.

**Figure 2:**
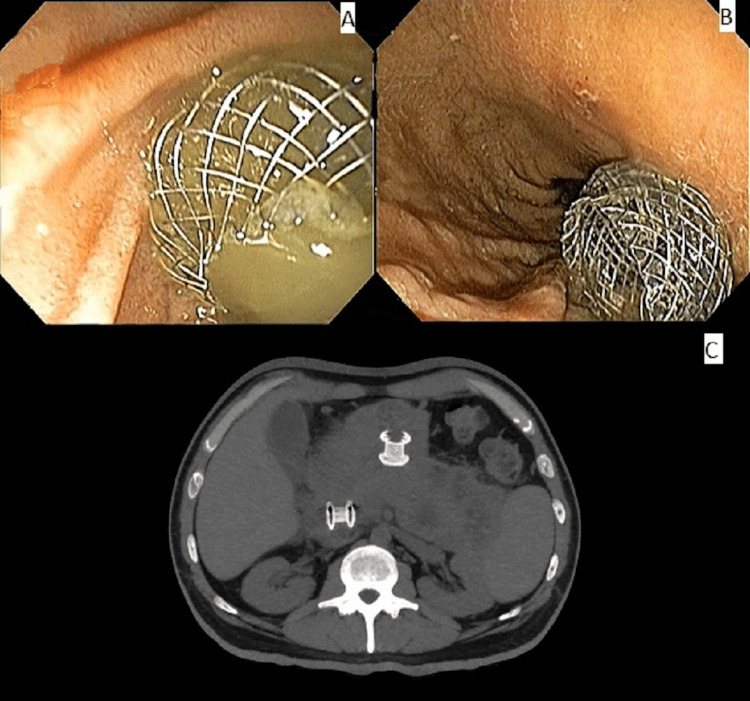
Endoscopic and computed tomography view of the two lumen-apposing metallic stents. (A) Transduodenal stent. (B) Transgastric stent. (C) Control abdominal computed tomography.

Simultaneous EUS drainage of both collections with two LAMS was performed. First, transduodenal drainage with a 15 mm × 10 mm metallic stent (diameter x extension) was done, followed by transgastric drainage with a 15 mm × 10 mm metallic stent. Both drainages promoted a large amount of thick purulent discharge. The total procedure time was 45 minutes (Figure 2, Video 1). The patient showed a notable clinical improvement with symptom relief. There was a substantial laboratory improvement as well, with a total bilirubin of 1.6 mg/dL and C-reactive protein of 9.5 mg/dL.

**Video 1:** Clinical case and endoscopic procedures. Clinical case and endoscopic procedures performed until patient discharge demonstrated by a didactic approach.

**Figure 3:**
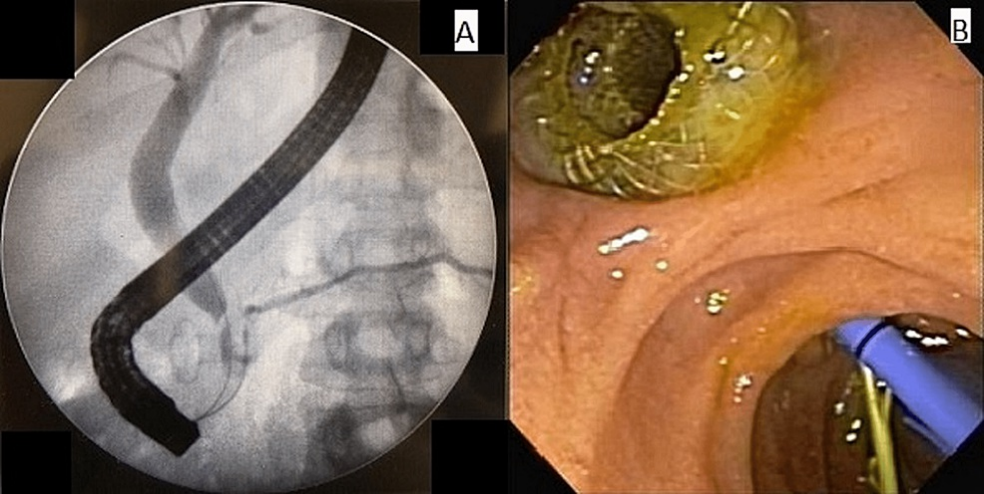
Fistula and its treatment by plastic stent deployment. (A) Proximal choledochal stenosis and fistula to residual collection. (B) Endoscopic view of the transduodenal metallic stent, biliary stent, and pancreatic stent.

Three weeks after drainage EGD, endoscopic retrograde cholangiopancreatography (ERCP) was performed which revealed a slight distal choledochal stenosis and leakage to the residual posteroinferior collection, characterizing a biliary fistula for the collection. Papillotomy was performed, with a 10 Fr × 10 cm plastic stent placement. The main pancreatic duct displayed an area of mild stenosis in the head/neck of the pancreas, but without contrast leakage. Pancreatic sphincterotomy was performed, with a 4 Fr × 9 cm plastic stent placement (Figure 3).

**Figure 4:**
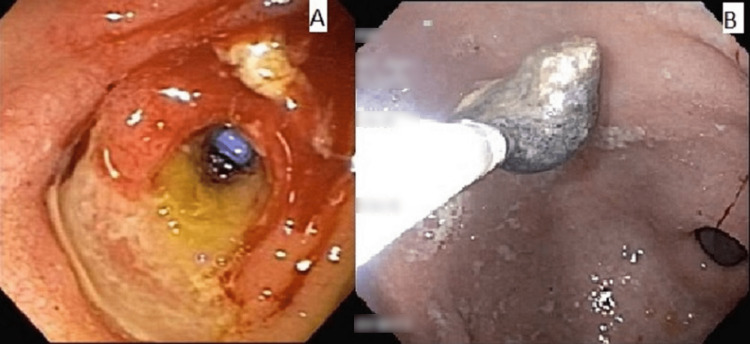
Transduodenal orifice after the removal of lumen-apposing metallic stents and direct endoscopic necrosectomy. (A) Transduodenal orifice after the removal of lumen-apposing metallic stents; biliary stent can be seen through the orifice. (B) Direct endoscopic necrosectomy.

The endoscopic review showed a residual posteroinferior periduodenal collection, through which it was possible to observe the biliary plastic prosthesis. We opted for the removal of the metallic stent. Access to the anterosuperior perigastric collection showed necrotic and fibrin content. Endoscopic lavage was performed using saline solution and hydrogen peroxide, followed by direct endoscopic necrosectomy (Figure 4). We opted for the removal of the metallic stent and passage of three double pig-tail 7 Fr × 7 cm transgastric plastic stents.

**Figure 5:**
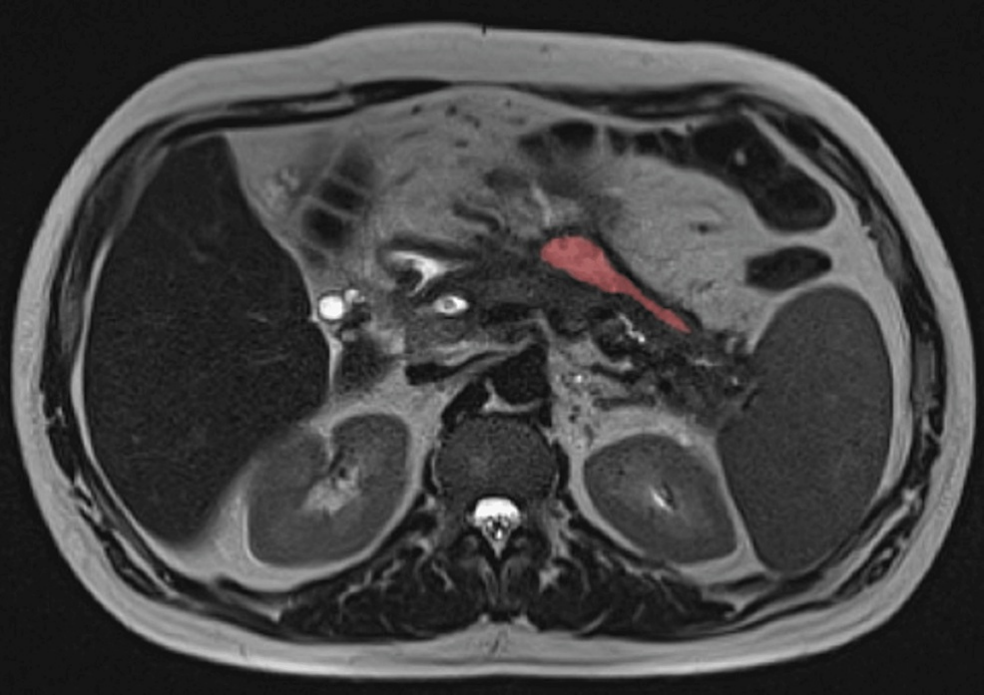
Small residual collection. Abdominal magnetic resonance imaging showing small residual collection (red).

Control MRI three months after the initial procedure demonstrated complete resolution of the posteroinferior collection and small residual anterosuperior collection, with an estimated volume of 13.9 mL (Figure 5). The patient remained asymptomatic during the six-month follow-up, performing his daily activities without restrictions, confirming technical and short-term clinical success.

We hereby describe the steps for the LAMS drainage. Fluoroscopic vision can be useful for understanding the exact location of the echoendoscope, particularly in the resolution of adverse events during the implantation of the stent that requires a rescue approach [8].

When assessing the deployment of two LAMS in the same procedure, it is essential to study the implantation site of each stent before starting the procedure. The most distal stent must be first implanted so that there is no risk of unintentional displacement of the first LAMS at the deployment of the second one. In the presented case, the transduodenal stent was the first, followed by the transgastric stent.

Once the implantation sites have been studied, a detailed EUS evaluation must be performed to identify four pivotal points: (1) the precise location and size of the collection; (2) the distance from the gastric and/or duodenal wall of the collection as a distance greater than 10-15 mm may contraindicate the procedure due to the extension of the prosthesis waist; (3) content, pseudocyst or WON - in the case of WON, estimate the amount of necrotic content, considering the use of a larger-diameter stent for better drainage and the eventual need for necrosectomy through the stent; (4) vascularization - evaluate the presence of pseudoaneurysms and avoid accidents by inadvertent puncture of interposed vessels between the collection and the gastric wall.

After the EUS evaluation, the drainage with the Hot-AxiosT system is summarized below with four steps performed for each collection:

1. Target access: deactivate the catheter lock and advance the catheter control center until the distal tip is visible on the ultrasound image; advance the catheter with pure cut current until it is 3-4 cm inside the target structure; and lock the catheter.

2. First flange deployment: remove the yellow safety clip, disable the stent lock, and move the implantation center to position 2 indicated on the accessory.

3. Stent retraction and alignment: disable the catheter lock and retract the catheter control center until a slight deformation of the flange against the collection wall is observed and then lock the catheter again.

4. Second flange deployment: deactivate the prosthesis lock and move the implantation center to the 4 position indicated on the accessory, releasing the second flange into the working channel; deactivate the catheter lock and push the catheter control center to couple the second flange to the gastric or duodenal wall under ultrasound or endoscopic vision.

The same steps must be performed for drainage with the second stent.

## Discussion

The treatment of symptomatic WON remains a challenge, especially in complex cases associated with multiple collections, and a large amount of necrotic content [9]. In complex cases, the association with percutaneous drainage may be necessary, and surgery is reserved for cases of failure or unavailability of minimally invasive techniques [10,11].

The development of minimally invasive surgery, whether or not associated with percutaneous drainage, represented a great advance in relation to open necrosectomy, demonstrating similar efficacy and lower complication rate in a clinical trial conducted by the Dutch Pancreatitis Study Group. Subsequently, the same group demonstrated that endoscopic treatment has the same efficacy as minimally invasive surgical treatment but with a shorter hospital stay and lower incidence of pancreatic fistula, favoring the endoscopic approach [12,13].

A systematic review and meta-analysis, including three randomized controlled trials and a total of 184 patients, was performed comparing the endoscopic approach versus minimally invasive surgery for WON. The analysis demonstrated no difference in mortality, although the endoscopic approach had fewer pancreatic fistula, enterocutaneous fistula, perforation, and multiple organ dysfunction after the intervention with statistical significance [14]. Furthermore, it was realized in a trial in the Netherlands that performed a long-term follow-up of seven years after endoscopic and surgical step-up approach demonstrates there is no superiority in reducing death or major complications in any group, although, after the initial six months, there were fewer pancreaticocutanous fistulas and reinterventions in the endoscopic group [15].

Despite the advantages of minimally invasive methods, the surgical approach continues to play an important role, especially in cases of clinical failure after minimally invasive drainage, unavailability or impossibility of EUS drainage, and in the management of caudal disconnected pancreatic duct syndrome, a complication associated with WON [16]. The disconnected pancreatic duct syndrome can be managed endoscopically with long-term transmural drainage with plastic stents, but there is a risk of stent migration, in addition to more rare complications such as a fracture of the stent, bleeding, and perforations. Thus, the surgical approach allows definitive treatment and becomes especially interesting in younger patients with a long life expectancy or in more complex cases [17,18].

There is a controversy regarding the type of stent that should be used routinely to drain pancreatic and peripancreatic collections. However, there is a predilection to use LAMS for WON drainage in cases where direct endoscopic necrosectomy can be necessary. Furthermore, in a recent meta-analysis, there were better clinical outcomes in the use of LAMS when compared to plastic stents [19,20]. We summarize some studies in Table 2.

**Table 2:** A summary of previous studies. MC: major complication; PF: pancreatic fistula; NOD: new-onset diabetes; MOF: multiple organ failure; CI: clinical improvement; WON: walled-off necrosis; LAMS: lumen-apposing metal stent; PS: plastic stent; AE: adverse events; QOL: quality of life

Study	Study design	Patient	Comparison	Number of patients	Outcomes	Follow-up time
van Santvoort et al. (2010) [12]	Clinical trial, multicenter	Necrotizing pancreatitis	Primary open necrosectomy and step-up approach	45 X 43	MC or death, PF, NOD, use of pancreatic enzymes, incisional hernia	Six months
van Brunschot et al. (2018) [13]	Clinical trial, multicenter	Necrotizing pancreatitis	Endoscopic and surgical step-up approach	51 X 47	MC or death, PF, exocrine and endocrine insufficiency, biliary strictures, wound infections, need for necrosectomy, the total number of interventions, length of hospital and ICU stay, costs, QOL	Six months
Bang et al. (2020) [14]	Meta-analysis	Infected necrotizing pancreatitis	Endoscopic and minimally invasive surgery	95 X 89	Mortality, MOF, enterocutaneous fistula, PF	NA
Onnekink et al. (2022) [15]	Clinical trial	Infected necrotizing pancreatitis after six months follow-up	Endoscopic and minimally invasive surgery	42 X 41	MC, death pancreaticocutaneous fistula, reinterventions, pancreatic insufficiency, and QOL	Seven years
Calo et al. (2022) [20]	Meta-analysis	WON drainage	LAMS and PS	493 X 514	Clinical improvement, AE, mortality	NA

Although the Hot-AxiosT system has advantages, it is expensive and not available in the routine of many hospitals in developing countries. Prospective randomized studies with cost-effectiveness analysis are necessary to understand the best indications for using the various stents available. Furthermore, it is essential to have a consonant multidisciplinary team with a surgeon in continuous contact with the endoscopist to choose the best-individualized approach for the patient.

## Conclusions

In the present report, the approach with two simultaneous LAMS was adopted due to the presence of two symptomatic non-communicating collections. If only one collection drainage was performed, the symptoms and the infectious condition would not be treated. The use of LAMS made it possible to approach both collections simultaneously with a procedure time of 45 minutes, achieving technical and short-term clinical success.

EUS drainage with two LAMS in the same procedure is feasible, safe, and effective; furthermore, it can be indicated in selected cases of symptomatic pancreatic collections in which drainage with only one stent is not sufficient.
